# Improving the diagnosis of heart failure in patients with atrial fibrillation

**DOI:** 10.1136/heartjnl-2020-318557

**Published:** 2021-03-10

**Authors:** Karina V Bunting, Simrat K Gill, Alice Sitch, Samir Mehta, Kieran O'Connor, Gregory YH Lip, Paulus Kirchhof, Victoria Y Strauss, Kazem Rahimi, A John Camm, Mary Stanbury, Michael Griffith, Jonathan N Townend, Georgios V Gkoutos, Andreas Karwath, Richard P Steeds, Dipak Kotecha, the RAte control Therapy Evaluation in permanent Atrial Fibrillation (RATE-AF) trial group

**Affiliations:** 1 Institute of Cardiovascular Sciences, University of Birmingham, Birmingham, UK; 2 Cardiology Department, University Hospitals Birmingham NHS Foundation Trust, Birmingham, UK; 3 NIHR Birmingham Biomedical Research Centre, University Hospitals Birmingham NHS Foundation Trust and University of Birmingham, Birmingham, UK; 4 Test Evaluation Research Group, Institute of Applied Health Research, University of Birmingham, Birmingham, UK; 5 University of Birmingham Clinical Trials Unit, Birmingham, UK; 6 Thrombosis Research Unit, Aalborg University, Aalborg, Denmark; 7 Liverpool Centre for Cardiovascular Science, University of Liverpool and Liverpool Heart and Chest Hospital NHS Foundation Trust, Liverpool, UK; 8 University Heart and Vascular Center, University Medical Center Hamburg-Eppendorf, Hamburg, Germany; 9 Medical Sciences Division, University of Oxford, Oxford, UK; 10 Deep Medicine, Nuffield Department of Women’s and Reproductive Health, University of Oxford, Oxford, UK; 11 NIHR Oxford Biomedical Research Centre, University of Oxford, Oxford, UK; 12 Cardiology Clinical Academic Group - Molecular & Clinical Sciences Institute, St George's University of London, London, UK; 13 Patient and Public Involvement Team, Birmingham, UK; 14 Institute of Cancer and Genomic Sciences, University of Birmingham, Birmingham, UK; 15 Health Data Research (HDR)-UK Midlands, Birmingham, UK

**Keywords:** atrial fibrillation, echocardiography, heart failure, diastolic, systolic

## Abstract

**Objective:**

To improve the echocardiographic assessment of heart failure in patients with atrial fibrillation (AF) by comparing conventional averaging of consecutive beats with an index-beat approach, whereby measurements are taken after two cycles with similar R-R interval.

**Methods:**

Transthoracic echocardiography was performed using a standardised and blinded protocol in patients enrolled in the RATE-AF (RAte control Therapy Evaluation in permanent Atrial Fibrillation) randomised trial. We compared reproducibility of the index-beat and conventional consecutive-beat methods to calculate left ventricular ejection fraction (LVEF), global longitudinal strain (GLS) and E/e’ (mitral E wave max/average diastolic tissue Doppler velocity), and assessed intraoperator/interoperator variability, time efficiency and validity against natriuretic peptides.

**Results:**

160 patients were included, 46% of whom were women, with a median age of 75 years (IQR 69–82) and a median heart rate of 100 beats per minute (IQR 86–112). The index-beat had the lowest within-beat coefficient of variation for LVEF (32%, vs 51% for 5 consecutive beats and 53% for 10 consecutive beats), GLS (26%, vs 43% and 42%) and E/e’ (25%, vs 41% and 41%). Intraoperator (n=50) and interoperator (n=18) reproducibility were both superior for index-beats and this method was quicker to perform (p<0.001): 35.4 s to measure E/e’ (95% CI 33.1 to 37.8) compared with 44.7 s for 5-beat (95% CI 41.8 to 47.5) and 98.1 s for 10-beat (95% CI 91.7 to 104.4) analyses. Using a single index-beat did not compromise the association of LVEF, GLS or E/e’ with natriuretic peptide levels.

**Conclusions:**

Compared with averaging of multiple beats in patients with AF, the index-beat approach improves reproducibility and saves time without a negative impact on validity, potentially improving the diagnosis and classification of heart failure in patients with AF.

## Introduction

The prevalence of atrial fibrillation (AF) is climbing,^1^ and around 50% of patients with AF have or will develop heart failure.^2 3^ Assessment of systolic and diastolic ventricular function using echocardiography is essential for AF management, including stroke risk stratification, choice of rate and rhythm control therapy, and the identification of heart failure.^4^ However, assessment of systolic and diastolic function is challenging in AF due to variable R-R intervals leading to beat-to-beat changes in left ventricular function. This may explain why the association of echocardiography parameters with adverse events is weaker in AF compared with sinus rhythm.^5^ In clinical practice, multiple beats are averaged despite a lack of any evidence base for this approach.^6^ Multiple beat acquisition and analysis is time-consuming and may not adequately compensate for the variable stroke volume and filling time seen in AF.^7^ To correctly classify heart failure in the context of AF and manage it appropriately, it is crucial to have accurate and reproducible measurements of systolic and diastolic function.^8^

The aim of this study was to systematically compare the reproducibility and efficiency of a more physiological approach to improve the assessment of heart failure in patients with AF. The index-beat method takes into account time-dependent processes involved in contractility and relaxation.^9^ We compared this approach with conventional averaging of 3 beats (clinical routine) and 5 beats and 10 beats (as recommended in guidelines), with complete blinding of observers. Unlike previous studies, there was no preselected exclusion of patients according to image quality.^10^ We hypothesised that the index-beat method would be more reproducible and time-efficient, facilitating better management of patients with AF and heart failure.

## Methods

### Patient population

Baseline echocardiograms were evaluated for all participants enrolled in the RATE-AF (RAte control Therapy Evaluation in permanent Atrial Fibrillation) randomised trial after obtaining written informed consent (NCT02391337). The trial received oversight from a trial steering committee and data monitoring committee with independent chairs. The rationale and methods for the RATE-AF trial have previously been published.^11^ In brief, outpatients aged 60 years or older with permanent AF and symptoms of heart failure (New York Heart Association class II or above) were randomised to digoxin or beta-blockers for rate control of AF. Permanent AF was defined as a physician’s decision for rate control and no plans for antiarrhythmic drugs or interventional rhythm control. Patients were excluded if their heart rate was <60 beats per minute or had prior evidence of second-degree or third-degree heart block. Other exclusion criteria were minimised to enable generalisable ‘real-world’ results. There were no exclusion criteria related to known heart failure or according to left ventricular ejection fraction (LVEF), apart from those with decompensated heart failure in the last 14 days, evidenced by the need for intravenous inotropes, vasodilators or diuretics.^11^ The trial was publicly funded by the UK National Institute for Health Research and the main results have been published.^10^

### Echocardiography protocol

AF was confirmed on 12-lead ECG. All patients then underwent transthoracic echocardiography using Philips EPIQ 7 and X5-1 transducer, by an experienced echocardiographer accredited with the British Society of Echocardiography. Patients were positioned in left lateral decubitus position and images were acquired during quiet respiration according to an echocardiography protocol ratified prior to the trial commencing.

A minimum of 30-beat loops were obtained of the apical two-chamber, three-chamber and four-chamber views. All images were optimised to maximise frame rate while ensuring all left ventricular segments were visible. A minimum of 30 traces of tissue Doppler imaging (TDI)-derived lateral and septal e’ were obtained and mitral inflow using pulse wave Doppler acquired at a sweep speed of 50 mm/s. The recorded images were given a unique identification number and all patient-identifiable features (including trial number) were removed, blinding the operator to patient, trial and clinical details.

All measurements were analysed offline by the same operator a minimum of 3 months after the scan date. To minimise selection bias, there were no predefined exclusions made on the quality of imaging; however, if image quality was insufficient to accurately obtain measurements, these images were excluded from analysis. All analyses were performed on Philips Q-Station (V.3.5; Philips Healthcare, Andover, Massachusetts). For LVEF, Simpson’s biplane LVEF was measured from the apical four-chamber and two-chamber views. Longitudinal strain was taken from the apical two-chamber, three-chamber and four-chamber views and then averaged to generate overall global longitudinal strain (GLS). The mean frame rate for GLS acquisition was 57 Hertz (SD 7.0). For E/e’, mitral valve peak E velocity was measured and then divided by the TDI-derived e’ (averaged from the lateral and septal walls).

### Index-beat and conventional averaging method

**Figure 1:**
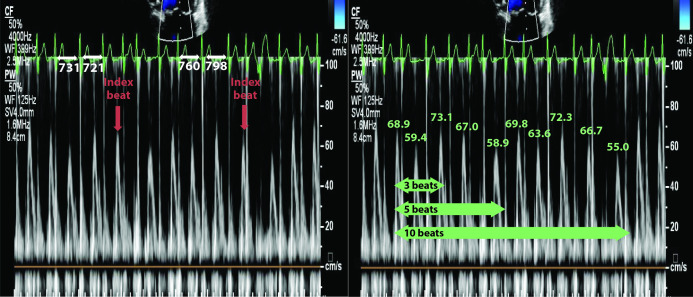
Index-beat approach versus conventional averaging of consecutive beats. Left panel: pulse wave mitral inflow Doppler using the index-beat method, with similar preceding and pre-preceding R-R intervals indicated by the red arrows. Values are the R-R interval length of the preceding cardiac cycle. Right panel: 3, 5 and 10 consecutive beats. Values are the peak E velocity measurement in cm/s.

The index-beat was identified as the cardiac cycle which followed a preceding and pre-preceding R-R interval of similar duration (within 60 ms of each other; figure 1). R-R intervals were measured using a calliper in the reporting software. The index-beats were selected consecutively from the beginning of each set of echocardiogram data to avoid selection bias. Conventional analysis involved averaging of 3, 5 and 10 consecutive cardiac cycles from a 10-beat data set.

### Intraoperator reproducibility

The same operator took a second set of 10 images to measure GLS and E/e’ (using the methods described above) with a single index-beat and an average of 3, 5 and 10 beats.

### Interoperator reproducibility and time comparison

A second accredited operator obtained images in 18 randomly selected patients to measure GLS and E/e’ using a single index-beat and average of 3, 5 and 10 beats. The operator was blinded to the previously recorded measurements as well as all clinical details of each echocardiogram. The time taken to select and measure E/e’ using a single index-beat and guideline-recommended 5 and 10 consecutive beats was measured, with time commencing from the first visualisation of Doppler images.

### Validity

All patients had N-terminal pro-B-type natriuretic peptide (NT-proBNP) measured on the same day as their echocardiogram using an Abbott Alinity platform. This was correlated with the parameters LVEF Simpson’s biplane, GLS and E/e’.

### Patient and public involvement

A patient and public involvement team were involved in the design and conduct of this study and assisted with the plain English summary.

### Statistical analysis

Summary results are presented as percentage, mean with SD, or median with IQR (displayed as 25th–75th quartiles). All echocardiographic measurements were transformed to their natural log value. A multilevel mixed effects linear regression model (adjusting for the random effects of the patient) was used to calculate the intraclass correlation coefficient (ICC) and derive the SD (σ), with 95% CI for the within-beat variability. Post-hoc analysis indicated >90% assurance that the sample size was sufficient for ICC comparison.^12^ The coefficient of variation (CV) within three index-beats and within each set of consecutive beats (3, 5 and 10) was calculated using the following formula: √exp(SD^2^)−1×100; 95% CIs were calculated for each CV. For intraoperator and interoperator reproducibility, Bland and Altman analysis was used to obtain the mean bias and limits of agreement, and the ICC with 95% CI was calculated at the patient level for each measurement method using a multilevel mixed effects linear regression model (adjusting for the random effects of the patient and the time the measurement was taken). The statistical comparison of time taken was performed using a two-tailed t-test accounting for unequal variance. The association with NT-proBNP was compared using Spearman’s r and univariate linear regression analysis. The difference in correlation coefficient between measuring on an index-beat and averaging 3, 5 and 10 consecutive beats was calculated using the method of Meng *et al*.^13^ Statistical analysis was performed using Stata V.14.2. A two-tailed p value of 0.05 was considered statistically significant.

## Results

One hundred and sixty patients were included, with a median age of 75 years (IQR 69–82), heart rate of 100 beats per minute at time of acquisition (86–112) and blood pressure of 134/84 mm Hg (123/76–148/93) (see table 1). The median LVEF was 59% (52–64), GLS −14% (−12 to −15) and E/e’ 9.4 (7.8–11.7); variables measured by consecutive beats are listed in online supplemental table 2. Image quality was insufficient for LVEF measurement in 18 patients (11.3%) and for GLS in 21 patients (13.1%), with strain assessment more challenging at higher heart rates. All patients had sufficient image quality to measure E/e’. Other echocardiogram parameters of interest are summarised in table 2. Online supplemental figure 1 depicts the flow diagram of the study population. A plain English summary of results is presented in online supplemental table 1.

10.1136/heartjnl-2020-318557.supp1 Supplementary data

**Table 1:** Baseline demographics

Characteristics	N=160
Age, median years (IQR)	75 (69–82)
Women, n (%)	74 (46)
Years in AF, mean years (SD)	3.8 (6)
Ethnicity: white British or Irish, n (%)	149 (93.1)
Ethnicity: black African, Caribbean or black British, n (%)	3 (1.9)
Ethnicity: Asian or Asian British, n (%)	8 (5.01)
Previous rhythm control, n (%)	23 (14)
Modified EHRA class 3 or 4, n (%)	77 (48)
Previous heart failure clinical diagnosis, n (%)	59 (37)
Signs of heart failure at randomisation, n (%)	84 (53)
NYHA class III or IV, n (%)	61 (38)
Previous myocardial infarction, n (%)	13 (8)
Previous stroke, n (%)	19 (12)
Previous TIA, n (%)	15 (9)
COPD, n (%)	29 (18)
Diabetes mellitus, n (%)	38 (24)
Heart rate, median bpm (IQR)	100 (86–112)
Systolic BP, median mm Hg (IQR)	134 (123–148)
Diastolic BP, median mm Hg (IQR)	84 (76–93)
Body mass index, median kg/m^2^ (IQR)	30 (26–34)
NT-proBNP, median pg/mL (IQR)	1057 (744–1522)
Estimated GFR, median mL/min (IQR)	67 (55–77)
Already receiving anticoagulant medication, n (%)	135 (84)
Antihypertensive medication, n (%)	116 (73)
Inhalers for airway disease, n (%)	40 (25)

AF, atrial fibrillation; BP, blood pressure; bpm, beats per minute; COPD, chronic obstructive pulmonary disorder; EHRA, European Heart Rhythm Association; GFR, glomerular filtration rate; NT-proBNP, N-terminal pro-B-type natriuretic peptide; NYHA, New York Heart Failure Association functional classification; TIA, transient ischaemic attack.

**Table 2:** Echocardiography parameters

Echocardiographic measurement	Baseline
Left ventricular end diastolic volume, median mL (IQR)	76 (57–99)
Left ventricular end systolic volume, median mL (IQR)	30 (22–42)
Stroke volume, median mL (IQR)	55 (45–64)
Left ventricular ejection fraction, median % (IQR)	59 (52–64)
Global longitudinal strain, median % (IQR)	−14 (−12 to −15)
Lateral s’, median cm/s (IQR)	6.7 (5.6–7.9)
Septal s’, median cm/s (IQR)	6.1 (5.1–7.2)
Average e’, median cm/s (IQR)	9.3 (8.1–10.9)
Mitral E velocity, median cm/s (IQR)	89.7 (77.1–102.8)
Mitral deceleration time, median ms (IQR)	212 (188–234)
Average E/e’, median (IQR)	9.4 (7.8–11.7)
Isovolumic relaxation time, median ms (IQR)	97 (89–108)
Pulmonary vein ratio, mean (SD)	0.7 (0.1)
Pulmonary vein deceleration time, median (IQR)	242 (223–258)
Left atrial volume indexed to BSA, median mL/m^2^ (IQR)	38 (32–49)
Left atrial ejection fraction, median % (IQR)	23 (15–33)
TAPSE mm, median (IQR)	18.7 (17.1–21.8)

BSA, body surface area; TAPSE, tricuspid annular plane systolic excursion.

### Within-beat reproducibility

**Figure 2:**
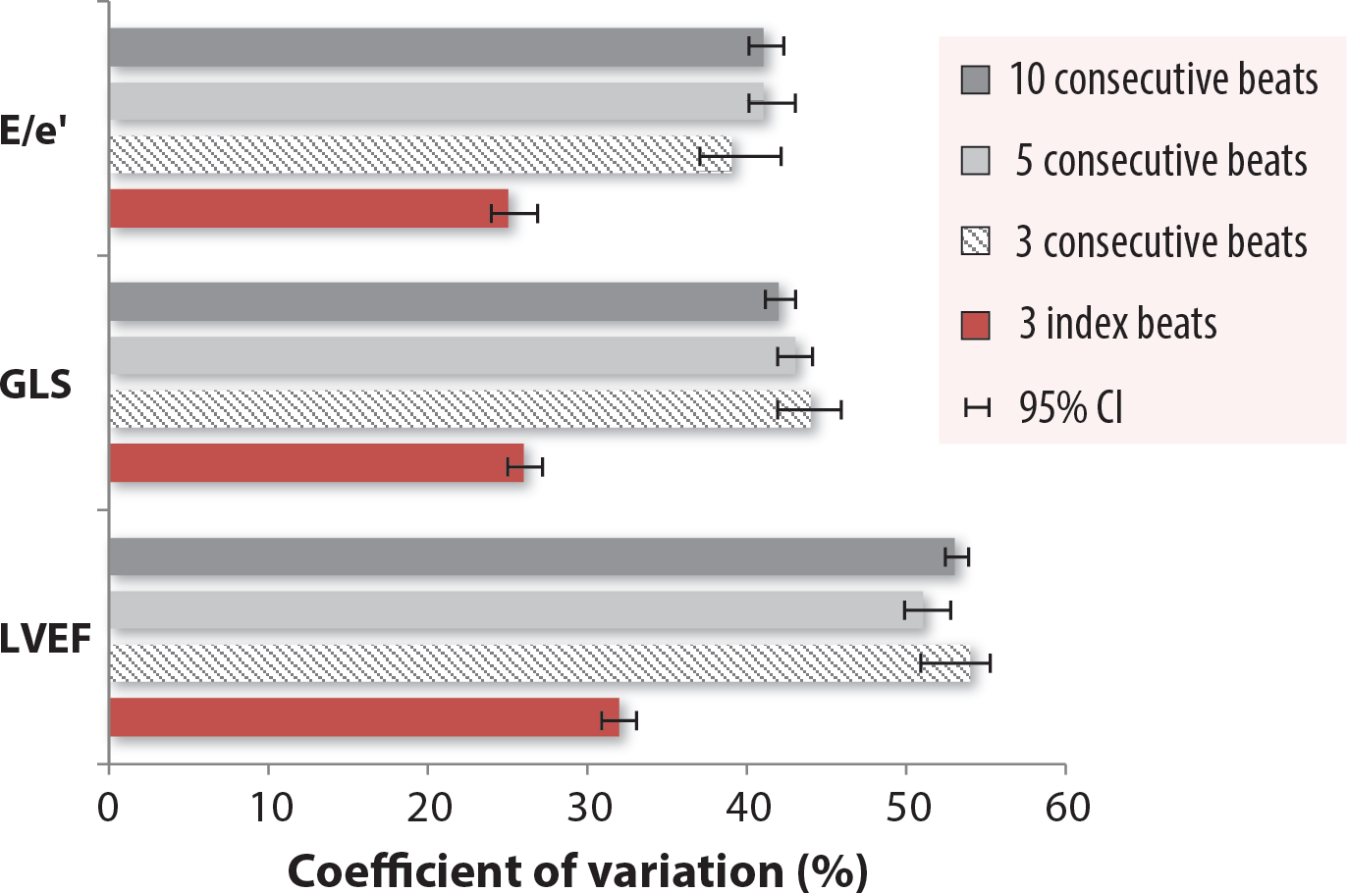
Comparison of the coefficient of variation of within-beat variability. Within-beat coefficient of variation for Simpson’s biplane LVEF, GLS and E/e’ between 3 index-beats, compared with 3, 5 and 10 consecutive beats with 95% CI. E/e’, mitral E wave max/average diastolic tissue Doppler velocity from the septal and lateral annulus; GLS, global longitudinal strain; LVEF, left ventricular ejection fraction.

**Figure 3:**
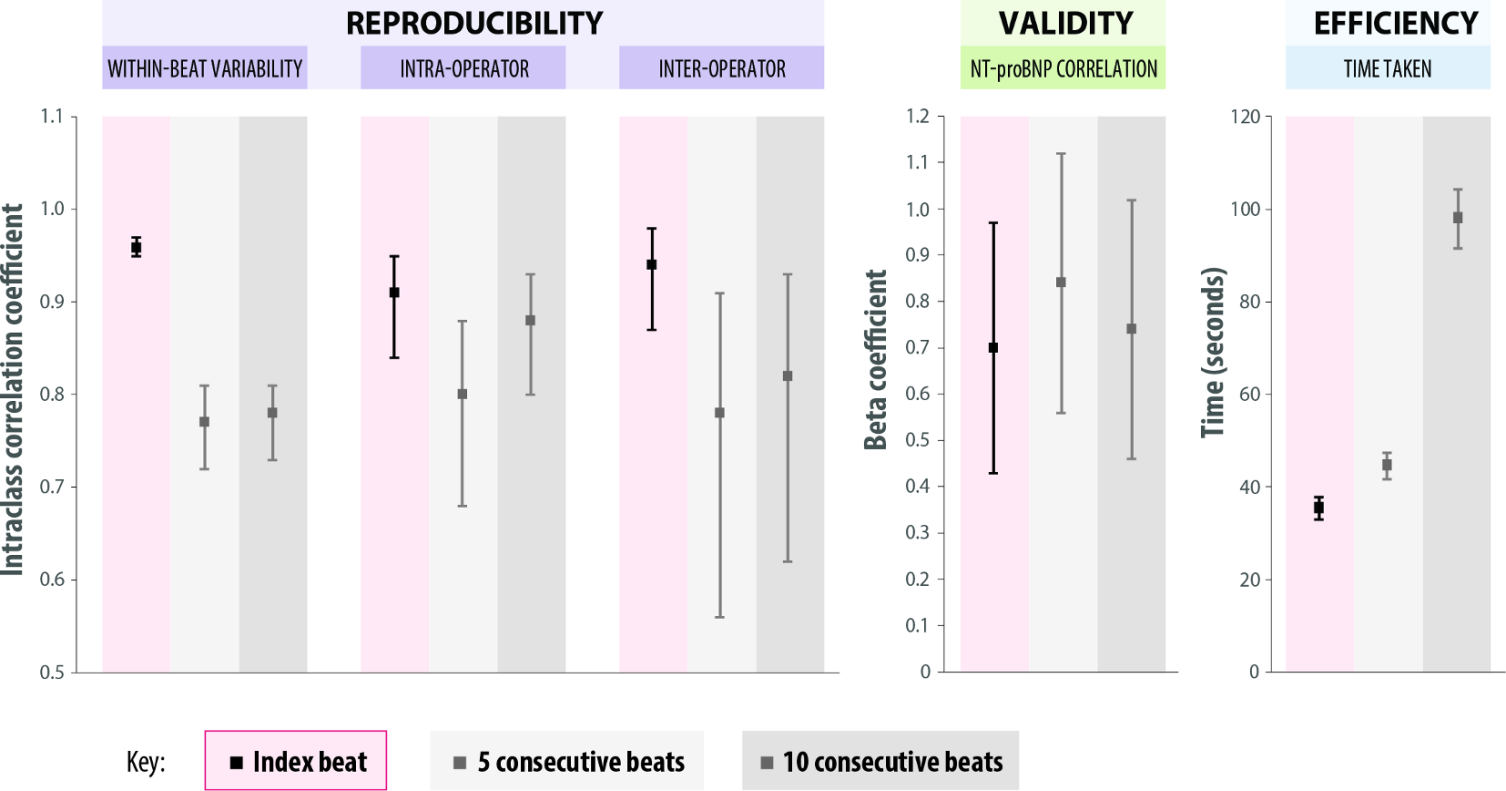
Reproducibility, validity and time efficiency of the index-beat approach for E/e’. Comparison of the index-beat method verses averaging of 5 and 10 consecutive beats for: (1) Reproducibility (ICC and 95% CI for within-beat, intra- and inter-operator variability); (2) Validity (beta coefficient for NT-proBNP with 95% CI); and (3) Time taken to measure E/e’ (mean seconds, with 95% CI). E/e’, mitral E wave max / average diastolic tissue Doppler velocity from the septal and lateral annulus; ICC, intraclass correlation coefficient; NT-proBNP, N-terminal pro-B-type natriuretic peptide.

The index-beat approach had less variability compared with conventional consecutive beat averaging for all comparisons of CV for LVEF, GLS and E/e’ (figure 2 and table 3). In all cases, the difference between the index-beat method and the variability of 3, 5 and 10 consecutive beats was statistically significant with no overlap of 95% CIs. Similarly, the index-beat approach demonstrated higher ICC values for LVEF, GLS and E/e’ (figure 3 and table 3).

**Table 3:** Within-beat variability of 3 index-beats versus 3, 5 and 10 consecutive beats

	LVEF Simpson’s biplane		GLS		E/e’	
	Coefficient of variation, % (95% CI)	Intraclass correlation coefficient (95% CI)	Coefficient of variation, % (95% CI)	Intraclass correlation coefficient (95% CI)	Coefficient of variation, % (95% CI)	Intraclass correlation coefficient (95% CI)
3 index-beats	32 (31 to 34)	0.94 (0.93 to 0.96)	26 (25 to 27)	0.88 (0.85 to 0.91)	25 (24 to 26)	0.96 (0.95 to 0.97)
3 consecutive beats	54 (52 to 57)	0.71 (0.64 to 0.77)	44 (42 to 46)	0.81 (0.76 to 0.85)	40 (37 to 41)	0.81 (0.76 to 0.85)
5 consecutive beats	51 (50 to 53)	0.76 (0.71 to 0.81)	43 (42 to 44)	0.82 (0.77 to 0.85)	41 (40 to 43)	0.77 (0.72 to 0.81)
10 consecutive beats	53 (52 to 54)	0.74 (0.69 to 0.79)	42 (41 to 43)	0.80 (0.75 to 0.84)	41 (41 to 42)	0.78 (0.73 to 0.81)

E/e', mitral E wave max/average diastolic tissue Doppler velocity from the septal and lateral annulus; GLS, global longitudinal strain; LVEF, left ventricular ejection fraction.

For LVEF, the index-beat approach had the smallest CV of 32% (95% CI 31 to 34) and strongest ICC of 0.94 (0.93 to 0.96), compared with 54% (52 to 57) and 0.71 (0.64 to 0.77) for 3 consecutive beats, 51% (50 to 53) and 0.76 (0.71 to 0.81) for 5 consecutive beats, and 53% (52 to 54) and 0.74 (0.69 to 0.79) for 10 consecutive beats. The mean RR1 of the index-beat (pre-preceding R-R interval) was 552 ms and the mean RR2 (preceding R-R interval) was 555 ms, with a mean RR1 to RR2 ratio of 1.00.

For GLS, the index-beat approach had the smallest CV of 26% (95% CI 25 to 27) and strongest ICC of 0.88 (0.85 to 0.91), compared with 44% (42 to 46) and 0.81 (0.76 to 0.85) for 3 consecutive beats, 43% (42 to 44) and 0.82 (0.77 to 0.85) for 5 consecutive beats, and 42% (41 to 43) and 0.80 (0.75 to 0.84) for 10 consecutive beats. The mean RR1 of the index-beat was 568 ms and the mean RR2 was 570 ms, with a mean RR1 to RR2 ratio of 1.09.

For E/e’, the index-beat approach had the smallest CV of 25% (95% CI 24 to 26) and strongest ICC of 0.96 (0.95 to 0.97), compared with 40% (37 to 41) and 0.81 (0.76 to 0.85) for the average of 3 beats, 41% (40 to 43) and 0.77 (0.72 to 0.81) for the average of 5 beats, and 41% (41 to 42) and 0.78 (0.73 to 0.81) for the average of 10 beats. The mean RR1 of the index-beat was 653 ms and the mean RR2 was 655 ms, with a mean RR1 to RR2 ratio of 1.00.

### Intraoperator and interoperator reproducibility

Intraoperator reproducibility of the index-beat method compared with consecutive beat averaging was assessed in 50 patients for E/e’ and GLS (with similar patient characteristics to the main cohort; online supplemental table 3). For E/e’, the index-beat had the smallest bias at −0.2 with similar limits of agreement to the average of 10 beats (−4.2 to 3.9) and the highest ICC of 0.91 (95% CI 0.84 to 0.95) (table 4 and online supplemental figure 2). Similar findings were seen for GLS, with the index-beat method having the smallest bias at −0.5 with narrow limits of agreement (−3.6 to 2.6) and the highest ICC of 0.82 (95% CI 0.72 to 0.90) (online supplemental table 4 and online supplemental figure 2).

**Table 4:** Intraoperator and interoperator reproducibility and time efficiency for E/e’

	Intraoperator reproducibility, n=50		Interoperator reproducibility, n=18		Time taken (s) to select and measure E/e’ (95% CI), n=18
	Bias (limits of agreement)	ICC (95% CI)	Bias (limits of agreement)	ICC (95% CI)	
Single index-beat	−0.2 (−4.2 to 3.9)	0.91 (0.84 to 0.95)	−0.3 (−2.9 to 2.2)	0.94 (0.87 to 0.98)	35.4 (33.1 to 37.8)
3 consecutive beats	−0.7 (−6.2 to 4.8)	0.74 (0.60 to 0.85)	−1.1 (−5.4 to 3.2)	0.83 (0.64 to 0.93)	Not performed
5 consecutive beats	−0.6 (−5.3 to 4.2)	0.80 (0.68 to 0.88)	−1.1 (−6.5 to 4.2)	0.78 (0.56 to 0.91)	44.7 (41.8 to 47.5)
10 consecutive beats	−0.4 (−4.2 to 3.4)	0.88 (0.80 to 0.93)	−0.9 (−6.1 to 4.2)	0.82 (0.62 to 0.93)	98.1 (91.7 to 104.4)

E/e’, mitral E wave max/average diastolic tissue Doppler velocity from the septal and lateral annulus; ICC, intraclass correlation coefficient.

Interoperator reproducibility was tested in 18 randomly selected patients for GLS and E/e’, with a new set of images taken by a second operator (with similar patient characteristics as seen in online supplemental table 3). The index-beat demonstrated only a small degree of bias for both GLS and E/e’. ICC values for the index-beat were 0.72 for GLS (95% CI 0.45 to 0.88) and 0.94 for E/e’ (95% CI 0.87 to 0.98) (table 4, online supplemental table 4 and supplemental figure 3). Comparison of the second operator’s assessment of E/e’ with measurements taken by the first operator (from a different set of images) demonstrated that the index-beat method had similar or higher levels of reproducibility than averaging 5 or 10 consecutive beats: Bland and Altman bias (limits of agreement) of −0.6 (−3.5 to 4.6), vs 0.8 for 5 beats (−3.3 to 4.9) and 0.4 for 10 beats (−2.7 to 3.6).

### Efficiency and validity of the index-beat method

The index-beat method took significantly less time to measure E/e’ (mean 35.4 s; 95% CI 33.1 to 37.8), compared with averaging 5 consecutive beats (44.7 s; 41.8 to 47.5; p<0.001) or 10 consecutive beats (98.1 s; 91.7 to 104.4; p<0.001) (figure 3 and table 4). Using the index-beat method saved 9.3 and 62.7 s, respectively (absolute difference in means).

Despite only using a single index-beat, there was no evidence that this impacted on the validity; there were no significant differences in the correlation of NT-proBNP with echocardiographic variables when comparing index-beats with conventional averaging (figure 3). For example, the correlation of LVEF with NT-proBNP was 0.11 using a single index-beat (p<0.001) vs 0.10 for 10 consecutive beats (p<0.001), with no significant difference between the two methods (p=0.84). Additional correlations for LVEF, GLS and E/e’ are presented in online supplemental table 5. Using the index-beat method led to reclassification of LVEF compared with 10-beat analysis (online supplemental table 6).

## Discussion

This study has demonstrated that the index-beat method produces more reproducible quantification of systolic and diastolic left ventricular function in patients with AF than conventional averaging of consecutive beats, while saving time and without compromising validity. The within-beat coefficient of variability and intraobserver/interobserver reproducibility were all favourable using the index-beat approach to imaging in patients who have a variable stroke volume and filling time. Using the index-beat method routinely in clinical practice has the potential to improve workflow and productivity, enhance the reliability of echocardiography, and provide more confidence in the diagnosis and classification of heart failure in patients with AF.

Heart failure is common in patients with AF, and accurate assessment of systolic and diastolic left ventricular function is essential for patient management.^4^ Although current guidelines recommend averaging 5–10 consecutive beats,^6 14 15^ this is based on consensus opinion and lacks reliable evidence.^16^ Measuring consecutive beats is time-consuming and the overall measurement obtained will vary according to which beats are selected, making reliability in clinical practice uncertain.^17 18^ AF is characterised by a loss of atrial contraction and so ventricular filling relies heavily on the length of the R-R interval, with variation in intervals leading to considerable challenges to achieve reproducible measurements.^19^ In addition to cycle length, stroke volume is critically dependent on preload, and this is also variable in the setting of AF.^9^

It is important to note the very high levels of variation we demonstrate using the guideline-recommended 10 consecutive-beat analysis; the CV of 41% for E/e’ should be considered unacceptable, highlighting the limitations of current practice in patients with AF. The influence of the R-R interval on contractility is believed to be caused by preload and uptake of calcium during the relaxation phase. The longer the R-R interval, the more time for calcium uptake and the greater the amount released in response to the sequential action potential, triggering a greater force of contraction.^20^ As ventricular filling and stroke volume for a particular beat are determined by the previous two R-R intervals, the index-beat method selects a cardiac cycle for analysis where the preceding and pre-preceding R-R interval are of similar duration. Hence, the real value of the index-beat method may be to achieve a more physiologically appropriate measurement. In this context, the end diastolic volume should be similar and so the contractility will also be similar, producing less variability between index-beats when assessing systolic function.^19 21 22^ The reliance on previous cycle lengths will also apply to GLS for detection of early myocardial dysfunction^17 23^ and diastolic filling indices such as E/e’.^24 25^

Intraoperator reproducibility for both GLS and E/e’ was shown to be highest when measuring on a single index-beat. Previous studies assessing strain rate have found a strong correlation between the index- beat and averages of 10 and 15 beats with high levels of agreement.^17 26^ We also demonstrated that the index-beat method has a high level of reproducibility between different operators, an important contribution to value within the clinical setting, enhancing the practicality and usefulness of serial scans.

### Strengths and limitations

A major concern with nearly all previous studies is the preselection of patients with ‘good echocardiography windows’.^7 27^ This is the first study to our knowledge in which the index-beat method has been interrogated in all patients with no pre-exclusions to image quality or heart rate, and all patients confirmed as being in AF at the time of echocardiography. The trial-based setting allowed us to ‘double-blind’ the imaging process; anonymised analyses were performed offline with a separate random code to the study identification number, and operators were blinded to patient details, clinical status and therapy. When compared with consecutive beats, our study showed no compromise in the correlation with NT-proBNP, a useful prognostic marker for both heart failure and coronary disease.^28^ However, this is clearly a limited analysis, and as the weak correlations between LVEF and NT-proBNP show, natriuretic peptide levels can be influenced by a variety of factors, including AF itself.^29^ When measured on an-index beat, LVEF was higher, on average, than using consecutive beats, and we also demonstrated that the index-beat led to reclassification of LVEF (online supplemental table 6). However, as we lack ‘ground truth’, further studies are warranted to compare the association of index-beat measurements with long-term clinical events in AF, and validation against invasive haemodynamic measurements. Our study was limited by non-simultaneous acquisition of Doppler and chamber images, and although this is currently standard practice globally there are single-beat options available for E/e’ that can also be valuable in AF.^30^ With pressure on echocardiography services increasing due to growing patient populations and wider indications, the index-beat method could increase the efficiency of echocardiography in AF; however, this was tested in a limited number of patients and only for E/e’. Qualitative assessment is also desirable to establish how to effectively introduce the index-beat approach in cardiology departments and the training required to aid clinical productivity.

## Conclusion

In a blinded analysis without preselection for image quality, assessing left ventricular function using the index-beat method provides a more reproducible and quicker method of assessing heart function in patients with AF. Pending independent validation, our results suggest that echocardiography departments should change to the index-beat method to diagnose and characterise heart failure in patients with AF.

**Key messages**

**What is already known on this subject?**

- Accurate assessment of ventricular function is essential to diagnose the type of heart failure in patients with atrial fibrillation (AF) and guide treatment strategies.
- However, measuring ventricular function in patients with AF is challenging due to the irregular cardiac cycle length.

**What might this study add?**

- The index-beat approach, whereby measurements of ventricular function are taken after two cycles with a similar R-R interval, was compared with conventional averaging of consecutive beats.
- We demonstrated that the index-beat approach was more reproducible, more time-efficient and did not compromise clinical validity against natriuretic peptide levels.

**How might this impact on clinical practice?**

- Pending external validation, use of the index-beat in clinical practice could improve the assessment of ventricular function, leading to more precise treatment strategies for patients with AF and the potential for fewer hospital admissions and better patient quality of life.

## Data Availability

Data sharing requests are required to meet the funder's policies; enquiries to be made to the corresponding author for Steering Committee review.
