# Supplementary material for: Improving the diagnosis of heart failure in patients with atrial fibrillation
Source: Heart. 2021 Mar 10;107(11):902–8. doi: 10.1136/heartjnl-2020-318557 (PMC8142420; doi:10.1136/heartjnl-2020-318557)
Supplement: Supplementary data [file heartjnl-2020-318557supp001.pdf]

Supplementary Table 1. Plain English Summary for patients

**About atrial fibrillation (AF)**
AF is a common heart condition that leads to an irregular and often rapid heart rate, which can make patients feel unwell and have more admissions to hospital. AF also causes 1 in 4 strokes and can reduce heart function over time resulting in a condition called heart failure.
**What was the purpose of this study?**
The assessment of heart function plays an important role in deciding what treatments a patient with AF should receive. To assess the function of the heart, patients are referred by their doctor to have an echocardiogram, which is an ultrasound scan of their heart. During the scan, multiple measurements are taken to determine how well the heart is pumping and relaxing. In patients with AF this is challenging because the heart rhythm is irregular causing measurements to vary between different heartbeats. Normally we average a number of heart beats, but this is time-consuming and may not be accurate in patients with AF.<br>In this study we compared the routine method of averaging measurements with a new method, based on taking measurements after heartbeats of similar length (called the index-beat method). This was carried out in patients taking part in a study to find out which heart rate control medication is better for patients, called the RATE-AF trial. The patients were aged 60 years and over with constant (permanent) AF who also had symptoms suggestive of heart strain or failure of the heart pump.
**Summary of the study findings**
Using the index-beat method caused less variability in measurements that assess how well the heart is pumping and relaxing. It also reduced the time taken to perform the scan when compared to averaging multiple beats. The new approach, although quicker, did not compromise how the measurements related to standard blood tests of heart function.
**Implications for patients**
It is essential that your heart function can be accurately assessed so that your doctor can prescribe the correct treatments for AF and heart failure. The index-beat method has been shown to improve the reliability of assessing heart function in AF patients and reduce the time taken for the heart scan. Its use in clinical practice could allow doctors to provide better treatment for AF patients, improving quality of life and preventing any worsening in health status.
**Patient support**
Patient information from the British Heart Foundation: https://www.bhf.org.uk/heart-health/conditions/atrial-fibrillation.<br>Education and support groups for patients and carers from the Heart Rhythm Alliance: https://www.heartrhythmalliance.org/.<br>Free smartphone and tablet apps for healthcare professionals and patients with AF from the European Society of Cardiology: www.escardio.org/af-apps.

Supplementary Table 2. Echocardiographic values measured using an average of three index-beats and average of 3, 5 and 10 consecutive bets

	**Average of 3 index-beats**	**Average of 3 beats**	**Average of 5 beats**	**Average of 10 beats**
LVEF, median % (IQR)	59 (52-64)	55 (50-60)	56 (50-60)	55 (49-59)
GLS, median % (IQR)	-14 (-12 to -15)	-13 (-11 to -15)	-13 (-11 to -15)	-13 (-11 to -14)
E/e', median (IQR)	9.4 (7.7-11.8)	9.5 (7.7-11.8)	9.4 (7.7-12.0)	9.5 (8.0-12.3)

E/e' = Mitral E wave max / average diastolic tissue Doppler velocity from the septal and lateral annulus; GLS= global longitudinal strain; LVEF = left ventricular ejection fraction

Supplementary Table 3: Baseline demographics of patients included in the intra and inter-operator reproducibility study

**Characteristic**	**All patients (n= 160)**	**Intra-operator reproducibility (n=50)**	**Inter-operator reproducibility (n=18)**
Age, median years (IQR)	75 (69-82)	79 (73-85)	74 (66-84)
Women, n (%)	74 (46%)	25 (50%)	8 (44%)
Years in AF, mean years (SD)	3.8 (6)	3.2 (6)	4.3 (8)
Modified EHRA class 3 or 4, n (%)	77 (48%)	22 (44%)	8 (44%)
Previous heart failure clinical diagnosis, n (%)	59 (37%)	13 (26%)	5 (28%)
Signs of heart failure at randomisation, n (%)	84 (53%)	27 (54%)	10 (56%)
NYHA class III/IV, n (%)	61 (38%)	21 (42%)	8 (44%)
Previous myocardial infarction, n (%)	13 (8%)	3 (6%)	2 (11%)
Previous stroke, n (%)	19 (12%)	5 (10%)	0 (0%)
Previous TIA, n (%)	15 (9%)	8 (16%)	0 (0%)
COPD, n (%)	29 (18%)	8 (16%)	3 (17%)
Diabetes mellitus, n (%)	38 (24%)	12 (24%)	5 (28%)
Previous rhythm control, n (%)	23 (14%)	7 (14%)	4 (22%)
Heart rate, median bpm (IQR)	96 (86-112)	96 (90-107)	99 (93-113)
Systolic BP, median mmHg (IQR)	134 (123-148)	134 (123-146)	132 (125-152)
Diastolic BP, median mmHg (IQR)	84 (76-93)	80 (75-86)	81 (76-86)
Body mass index, median kg/m^2 (IQR)	30 (26-34)	29 (27-33)	29 (26-34)
NT-proBNP, median pg/mL (IQR)	1057 (744-1522)	1062 (738-1480)	1116 (761-1192)
Estimated GFR, median mL/min (IQR)	67 (55-77)	67 (55-73)	70 (52-83)
Anticoagulant medication, n (%)	135 (84%)	40 (80%)	14 (78%)
Antiplatelet medication, n (%)	9 (6%)	2 (4%)	1 (6%)
Antihypertensive medication, n (%)	116 (73%)	37 (74%)	14 (78%)
Inhalers for airways disease, n (%)	40 (25%)	7 (14%)	2 (11%)

AF= atrial fibrillation; BP= blood pressure; COPD= chronic obstructive pulmonary disorder; EHRA= European Heart Rhythm Association; GFR= glomerular filtration rate; NT-proBNP= N-terminal pro-brain natriuretic peptide; NYHA= New York Heart Failure Association functional classification; TIA= transient ischaemic attack.

Supplementary Table 4. Intra and inter-operator reproducibility of GLS.

Reproducibility	Single index beat	3 consecutive beats	5 consecutive beats	10 consecutive beats
**Intra-operator (n=50)**				
Bias (limits of agreement)	-0.5 (-3.6 to 2.6)	-1.1 (-4.8 to 2.7)	-1.1 (-4.4 to 2.2)	-1.0 (-4.0 to 2.0)
ICC (95% CI)	0.82 (0.72 to 0.90)	0.75 (0.61 to 0.85)	0.76 (0.63 to 0.86)	0.80 (0.68 to 0.88)
**Inter-operator (n=18)**				
Bias (limits of agreement)	-0.3 (-5.5 to 5.0)	-0.8 (-5.7 to 4.1)	-0.7 (-5.5 to 4.1)	-0.7 (-5.3 to 3.9)
ICC (95% CI)	0.72 (0.45 to 0.88)	0.74 (0.50 to 0.90)	0.75 (0.50 to 0.90)	0.77 (0.54 to 0.91)

Intra and inter-operator reproducibility of GLS when using a single index-beat verses the average of 3, 5 and 10 beats. Displayed as Bland and Altman analysis (bias and limits of agreement) and the intra-class correlation coefficient derived from the mixed effects multi-level linear regression model adjusting for the patient and measurement time.

Supplementary Table 5: Validity of a single index beat verses averaging consecutive beats when correlating with NT-proBNP.

Validity	Single index Beat	3 consecutive beats	5 consecutive beats	10 consecutive beats
**LVEF (n=142)**				
β coefficient	-1.04	-1.01	-1.01	-1.05
Regression R^2 (model p)	0.11 (p<0.001)	0.10 (p<0.001)	0.10 (p<0.001)	0.10 (p<0.001)
Difference from index beat Z test (p-value)	-	-0.34 (p=0.73)	-0.28 (p=0.78)	-0.20 (p=0.84)
**GLS (n=139)**				
β coefficient	0.07	0.06	0.07	0.06
Regression R^2 (model p)	0.03 (p=0.033)	0.03 (p=0.031)	0.04 (p=0.020)	0.03 (p=0.034)
Difference from index beat Z test (p-value)	-	-0.04 (p=0.97)	-0.31 (p=0.75)	-0.03 (p=0.98)
**E/e' (n=160)**				
β coefficient	0.69	0.79	0.84	0.74
Regression R^2 (model p)	0.13 (p=<0.001)	0.16 (p=<0.001)	0.18 (p=<0.001)	0.15 (p=<0.001)
Difference from index beat Z test (p-value)	-	-1.08 (p=0.28)	-1.91 (p=0.06)	-1.02 (p=0.31)

Univariate linear regression analysis to show the correlation between LVEF Simpson's biplane, GLS and E/e' measured using a single index-beat verses the average of 3, 5 and 10 consecutive beats. The beta coefficient describes the change in echocardiographic parameter for a single unit increase in NT-proBNP. A test dependent correlation coefficient (CorCor) was used to assess the difference in correlation coefficient when measuring on a single index beat verses averaging consecutive beats.

LVEF= left ventricular ejection fraction; GLS= global longitudinal strain; NT-proBNP= N-terminal B-type natriuretic peptide

Supplementary Table 6: Re-classification using the index-beat approach

		**LVEF (%) measured using a single index-beat**					
Classification of LVEF		Quintile 1 45% (35-49%)	Quintile 2 54% (52-55%)	Quintile 3 59% (58-60)	Quintile 4 63% (62-64%)	Quintile 5 68% (66-69%)	Total
**LVEF (%) measured by the average of 10 consecutive beats**	Quintile 1 41% (32-45%)	22	6	1	1		30
	Quintile 2 51% (40-53%)	7	7	5	5	3	27
	Quintile 3 55% (55-57%)		11	7	7	5	30
	Quintile 4 58% (58-59%)		3	11	6	7	27
	Quintile 5 62% (61-63%)		2	5	8	13	28
Total		29	29	29	27	28	142

Key:	Lower LVEF quintile when reclassified using the index-beat method	No difference in LVEF quintile	Higher LVEF quintile when reclassified using the index-beat method
Number (%)	47 (33%)	55 (38%)	40 (28%)

		**E/e' measured using a single index-beat**					
Classification of E/e'		Quintile 1 6.2 (5.9-6.8)	Quintile 2 8.1 (7.8-8.3)	Quintile 3 9.5 (9.0-9.9)	Quintile 4 11.1 (10.5-11.5)	Quintile 5 14.9 (13.7-19.1)	Total
**E/e' measured by the average of 10 consecutive beats**	Quintile 1 6.4 (6.0-7.1)	26	6				32
	Quintile 2 8.5 (8.1-8.8)	4	21	10			35
	Quintile 3 9.6 (9.4-10.1)	1	7	13	8		29
	Quintile 4 11.6 (11.1-12.4)		1	7	19	5	32
	Quintile 5 14.7 (14.1-17.3)				4	27	31
Total		31	35	30	31	32	159

Key:	Lower E/e' quintile when reclassified using the index-beat method	No difference in E/e' quintile	Higher E/e' quintile when reclassified using the index-beat method
Number (%)	24 (15%)	106 (67%)	29 (18%)

E/e' = Mitral E wave max / average diastolic tissue Doppler velocity from the septal and lateral annulus; LVEF = left ventricular ejection fraction

Using the average of 10 consecutive beats and a single index-beat for each individual patient, LVEF and E/e' were categorised into quintiles (values for each quintile report the median value and interquartile range using that method). Reclassification of LVEF quintile is indicated by the coloured boxes, with the reference being the conventional 10-beat analysis. The two approaches show only moderate agreement for LVEF, with a weighted Cohen's Kappa of 0.48 (Z=8.33; p<0.001) and 2-way random-effects intraclass correlation coefficient of 0.66 (F=4.94; p<0.001); respective values for E/e' are 0.78 (Z=14.3; p<0.001) and 0.91 (F=20.60; p<0.001) in contrast suggest a strong agreement between the two approaches.

Supplementary Figure 1. Flow diagram to show the study population

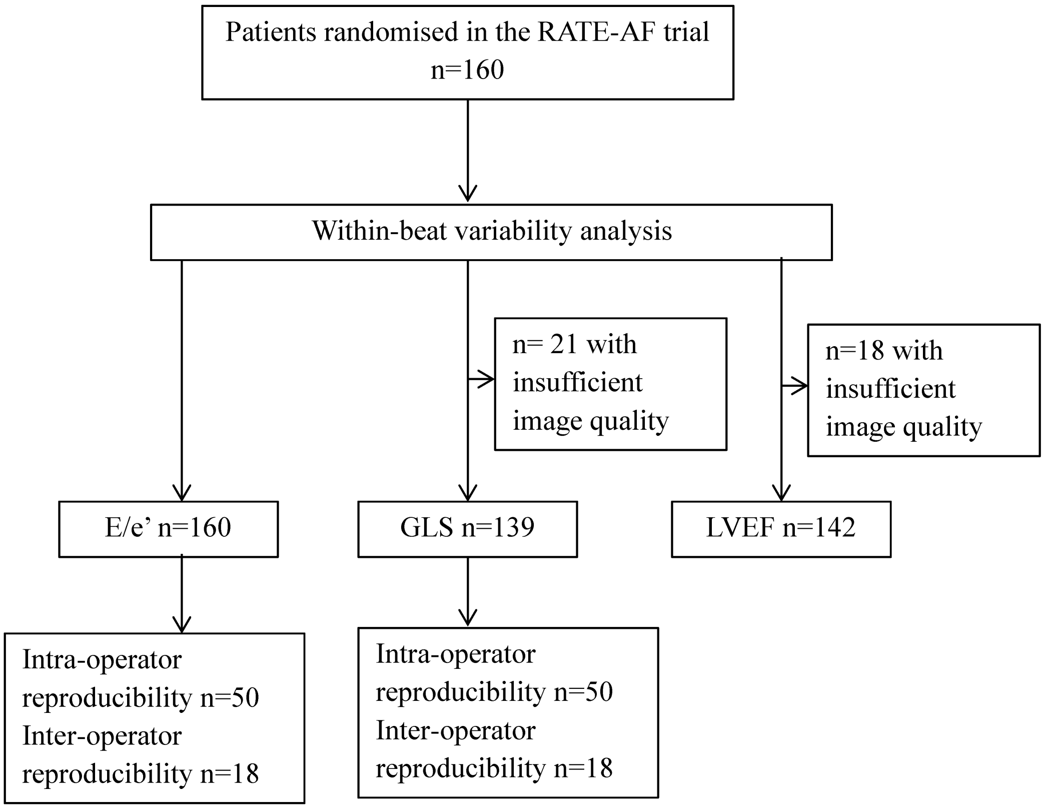

E/e'= Mitral E wave max / average diastolic tissue Doppler velocity from the septal and lateral annulus; GLS= global longitudinal strain; LVEF = left ventricular ejection fraction; RATE-AF= RAte control Therapy Evaluation in permanent Atrial Fibrillation

Supplementary Figure 2. Bland and Altman plots to show GLS and E/e' intra-operator reproducibility in 50 patients

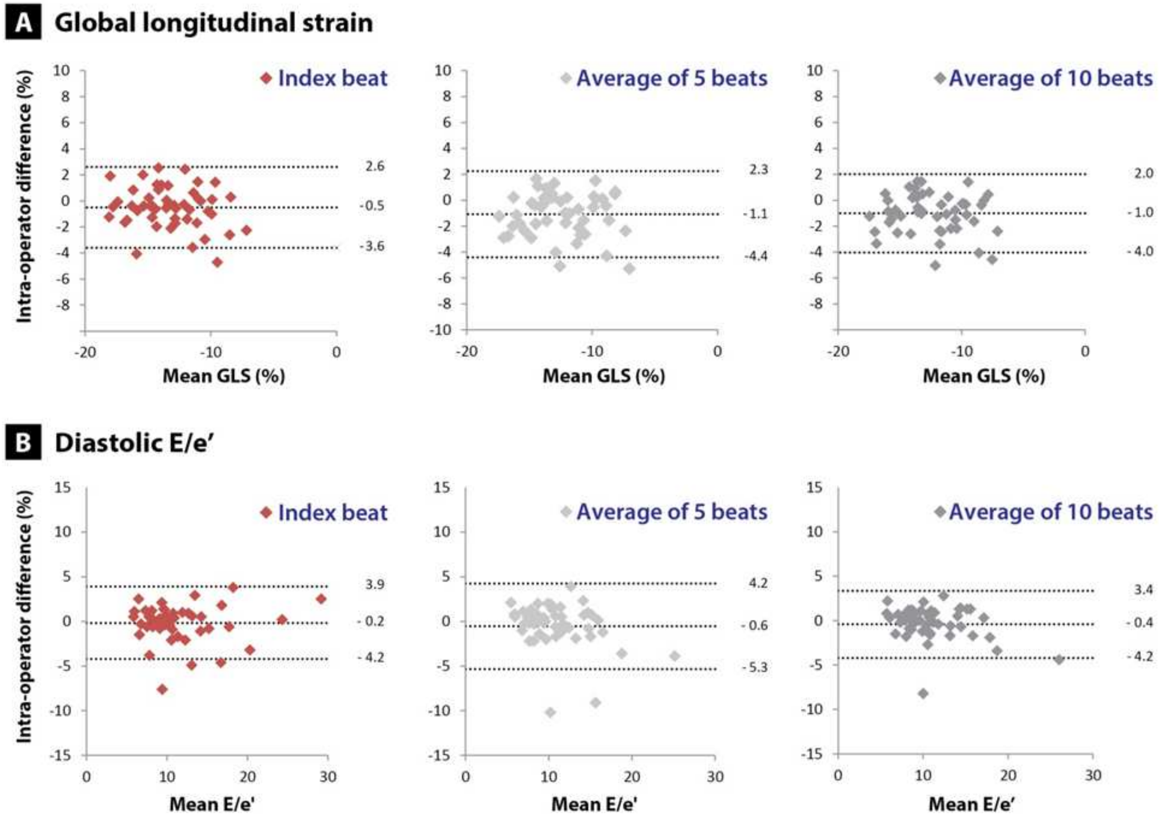

(A) GLS and (B) E/e' when measured by the same operator, using a single index-beat versus the average of 5 and 10 consecutive beats. E/e' = Mitral E wave max / average diastolic tissue Doppler velocity from the septal and lateral annulus; GLS = global longitudinal strain .

Supplementary Figure 3. Bland and Altman plots to show GLS and E/e' inter-operator reproducibility in 18 patients

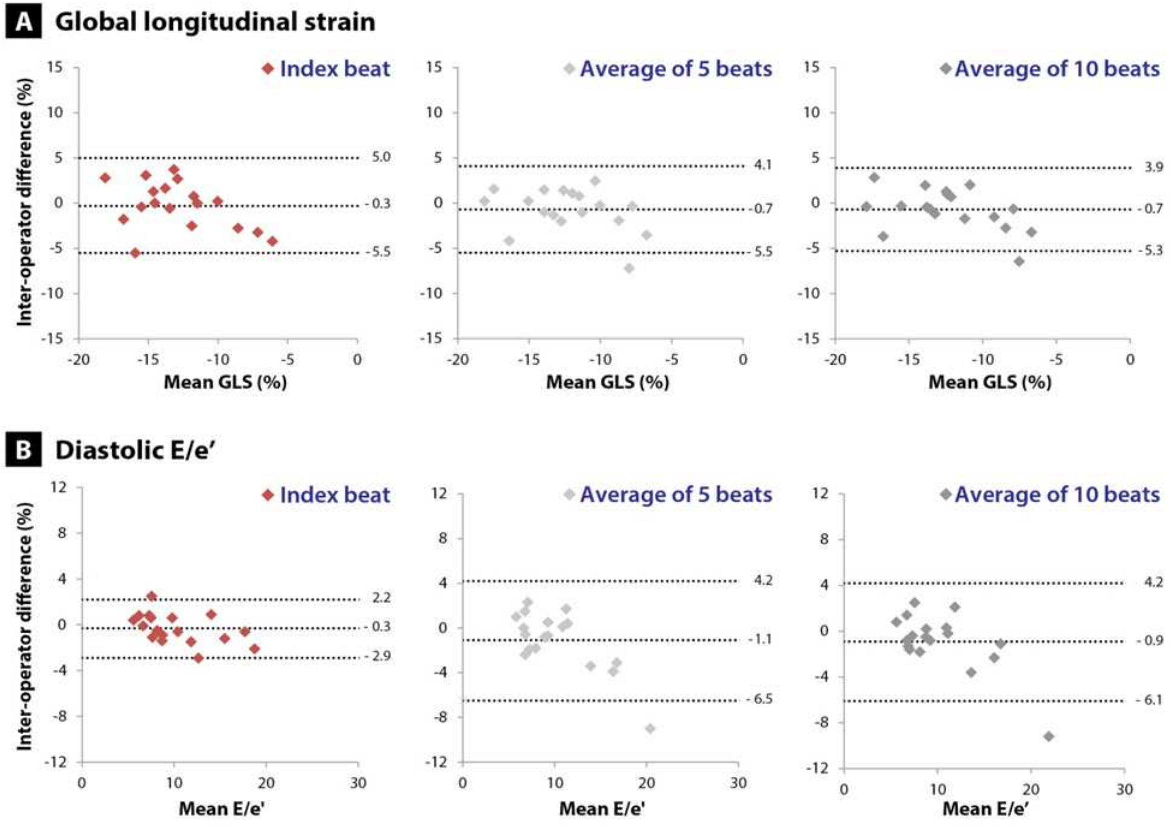

(A) GLS and (B) E/e' when measured by a second operator, using a single index-beat versus the average of 5 and 10 consecutive beats. E/e'= Mitral E wave max / average diastolic tissue Doppler velocity from the septal and lateral annulus; GLS = global longitudinal strain.
